# Reduction of coercive measures under routine conditions in psychiatric hospitals 2004–2019: Strong effects in old age psychiatry, much less in general psychiatry

**DOI:** 10.1192/j.eurpsy.2020.104

**Published:** 2020-11-20

**Authors:** Tilman Steinert, Sophie Hirsch, Rita Goebel, Brendan Snellgrove, Erich Flammer

**Affiliations:** 1 Clinic for Psychiatry I, Ulm University, Ulm, Germany; 2 Centres for Psychiatry Suedwuerttemberg, Ravensburg, Germany; 3 Department of Neurology, University of Tübingen, Tübingen, Germany

**Keywords:** Coercion, epidemiology, general psychiatry, old age psychiatry

## Abstract

**Background:**

Many interventions to reduce the use of seclusion and restraint have been suggested in the last decades. Evidence-based interventions in old age psychiatry are different from those in general psychiatry. A common database for psychiatric hospitals introduced in 2004 allowed to examine the use of seclusion and restraint over 16 years under routine conditions.

**Methods:**

A registry for coercive measures in the Federal State of Baden-Wuerttemberg has been available since 2015, and comprises all 32 hospitals licensed to admit involuntary patients. A study group had collected data prospectively since 2004 from a subsample of these hospitals. We analyzed the mean percentage of patients subjected to coercive measures and the mean cumulative duration of these interventions in ICD-10 diagnostic groups in psychiatric hospitals from 2004 to 2019 among a total of 1,038,239 admissions.

**Results:**

The proportion of cases affected by coercive measures dropped significantly from 28.4 to 10.5% in patients with ICD-10 F0 disorders, while rates in patients with other diagnoses decreased insignificantly from 7.0 to 5.4%. The cumulated duration of coercive measures per affected case also dropped significantly among patients with F0 disorders, while changes in patients with other diagnoses remained insiginficant.

**Conclusions:**

The use of coercive measures in patients with organic disorders could be reduced by about 50% in a State of 11 million inhabitants within 15 years, while in contrast no substantial reduction occurred among all other diagnostic groups. Specific interventions to reduce coercive interventions seem to be particularly successful for this patient group.

## Introduction

Reducing the use of coercive interventions in in-patient psychiatry has been a major issue of interest in many countries. A considerable range of interventions has proved some efficacy in controlled studies [1]. However, even for highly favoured intervention such as de-escalation training and joint crisis plans evidence is still inconsistent. [2–4]. Evidence of effectiveness under routine conditions is scarce. More recently, complex interventions combining interventions with different targets have been developed, namely the Safewards Model in the United Kingdom [5–8] and the Six Core Strategies in the United States [9–11]. In Germany, a seminal cornerstone was the publication of evidence- and consensus-based guidelines for the prevention of coercive measures in 2018 [12,13]. Many of the suggested interventions have been previously proposed in a working group encompassing about half of the psychiatric hospitals in the Federal State of Baden-Wuerttemberg in awareness workshops twice a year since 2000 [14], relying on international experience. In addition, a common database was created with common definitions of coercive measures. Beginning in 2004 and funded by a Federal research project, the implementation of electronic charts in routine care allowed collecting and analyzing patient-related data without the loss of paper sheets and time-consuming data transfer. Thereby, epidemiological data on the frequency and duration of coercive measures, first of all mechanical restraint and seclusion, became available and allowed for hospital comparisons [14]. We defined outcome variables that could be assigned not only to institutions but also to diagnosis groups according to ICD-10 [14]. This system of data recording and analysis was adopted in the following years in Switzerland [15] and in the Netherlands [16]. Since 2015, this documentation is mandatory for all 32 psychiatric hospitals of the Federal State that are licensed to treat involuntarily admitted patients.

In our first cross-sectional study covering 10 hospitals in 2004, 28.0% of 36,690 admissions with ICD-10 F0 (organic disorders), but only 7.0% of all other disorders, had been subjected to coercive measures [14]. Patients with these disorders had by far the highest burden of coercion, mostly due to the risk of falls. Most patients with organic disorders are patients with dementia and either delirium or severe behavioral disorders frequently combined with somatic multimorbidity and problems of care who are admitted if nursing homes or relatives are no longer able to cope with behaviour difficulties. Typically, these patients are treated in more or less specialized geriatric units within psychiatric hospitals. The reasons for the use of coercive interventions were frequently mixed at that time. They were predominantly used in prevention of falls, but also to prevent patients from removing medical devices, such as tubes, and aggressive behavior toward others. The reasons are more or less the same as those reported from other countries [17]. Within the period since 2004, recommendations to reduce coercion in old age psychiatry have differed considerably from those in general psychiatry. While interventions in general psychiatry focus particularly on respect for the patients’autonomy, de-escalating communication, and assessment of violence risk [12], old age psychiatry is more focused on risk of falls [18] and technical devices [19].

Assuming that there has been some willingness to change clinical practice and attitudes toward coercive measures in the hospitals according to the suggestions of the aforementioned workshops, this longitudinal database allows examining the following study questions:

1. Did the use of coercive interventions change under conditions of routine care in a region covering 11 million inhabitants in the course of 15 years?
2. Were there differences between people with organic disorders and other diagnostic groups?

## Methods

### Data sources

Data collection was initiated in the early years after 2000, primarily on initiative of clinical directors. This was then supported by a grant from the Federal State of Baden-Wuerttemberg in 2004. At that time, we developed common definitions of coercive interventions to be applied in all participating hospitals, and a system of data collecting and reporting. We decided to determine the mean percentage of admissions subjected to any kind of individual mechanical coercion (mechanical or physical restraint, and seclusion) and the mean total duration of these interventions per affected case. Involuntary medication has been recorded only since the beginning of 2015 due to changes of legislation. The figures are low compared with seclusion and restraint [20] and were not considered here. Until 2014, each hospital collected and analyzed data individually. The methods have been described in detail in previous publications [14].

In 2015, a new Mental Health Law was introduced in the German Federal State of Baden-Wuerttemberg following a Supreme Court decision. It comprised a unique feature, obliging all 32 public psychiatric hospitals (including those who had collected these kinds of data previously and thus had yielded evidence on feasibility of this procedure) to collect data on seclusion, restraint, emergency medication, and involuntary medication. Since then, it has been mandatory for all psychiatric hospitals to supply these data to a central registry. In contrast to the previous procedure, hospitals do not conduct own analyses but report data on each single intervention to the registry. Considering the highly sensitive personal data, this procedure requires special demands on data privacy and data security. An online platform was set up after detailed consultation with the State Data Privacy and Data Security Officer and his final approval. The platform serves for both uploading data by the institutions and downloading data by the evaluation office. Data privacy is ascertained by a double and irreversible pseudonymisation carried out by different institutions and by use of passwords. Thus, the identification of individual persons is not possible, that is, the data are anonymized. For each coercive intervention, the dataset contains the kind of intervention as defined by a codebook, its legal basis, the duration, the patient’s gender, and the ICD-10 principal group. For all hospitals, numbers of admissions with respect to diagnoses and percentages of involuntary admissions according to different laws are available [20].

### Ethics

The Ethics Committee of Ulm University waived the requirement for ethics approval as approval is not required for studies analyzing anonymized data, in accordance with national legislation and institutional requirements.

### Definitions

Comparisons of longitudinal data as presented here only make sense if definitions of coercive interventions are identical: (a) across hospitals and (b) over the time. Fortunately, this was the case, since hospitals continued to use the same definitions as agreed upon in 2004 and these were the blueprint for the mandatory codebook published by the Ministry of Social Welfare in 2015. This is particularly important for geriatric psychiatry, where it is sometimes difficult to reach a consensus on where, in contrast to milder forms of confinement, coercion begins. We used a rather clear and wide definition of restraint: all kinds of freedom-restricting devices should be counted as mechanical restraint, encompassing not only belts in beds, but also (undivided) bedrails, movement-restricting blankets, tables attached to a chair, and other devices, as far as they restrict free movement. Physical restraint (staff holding a person over some time by force) is uncommon in geriatric psychiatry in Germany. Seclusion was defined according to suggestions in the literature [21] as locking the person in a scarcely furnitured room (mostly only with a matress and toilet) without presence of staff. Involuntary medication is rare as a consequence of the high legal threshold [22] and the well-known considerable side effects among people with dementia. It was not systematically recorded before 2015 and is therefore not considered here.

### Diagnoses

The dataset contains for each case the principal ICD-10 diagnosis in the form of the first digit (F0–F9), as provided to health insurance companies. Secondary diagnoses are not available. Data from forensic psychiatric units are also available [23] but have been excluded here. For the purpose of this analysis, we separated F0 cases (as a proxy for dementia) from all other cases (F1–F9; as a proxy for general psychiatry). Distribution of diagnoses and coercive interventions in the diagnostic groups F1–F9 have been analyzed in previous publications [14,20].

The diagnostic group ICD-10 F0 is mostly but not exactly identical with dementia and delirium. It is possible that a few younger patients with organic brain disorders might be included. However, these disorders are rarely a reason for hospitalization in in-patient psychiatry.

### Analyses

We analyzed aggregated data on the proportion of patients affected by coercive interventions, number and duration of coercive interventions and diagnoses. Data were aggregated on hospital level. We calculated means and standard deviations across hospitals for each year. To assess whether the proportion of cases involving coercive interventions and whether the cumulated duration of coercive interventions showed significant changes over time, linear regression analyses were performed with time as predictor and the proportion of patients affected by coercive interventions and the cumulated duration of coercive interventions per affected case as dependent variables. Linearity was assessed by visual inspection of the plots of observed versus predicted values. The regression analyses were performed for F0 and all other diagnoses seperately. Homoscedasticity was assessed by visual inspection of the plots of residuals versus time. The normal distribution of residuals was tested with Kolmogorov–Smirnov tests. In order to adjust for multiple testing, Bonferroni adjustments were made for testing the overall statistical significance of the models as well as for testing the statistical significance of the regression coefficients. Overall statistical significance was tested with analysis of variance, and statistical significance of the coefficients was tested with *t* tests.

Furthermore, we examined whether the sample of those hospitals that participated in data recording on a voluntary basis before the start of the mandatory registry in 2015 was representative. To this end, we analyzed the data of the subset of those 18 hospitals that had participated in 2014 on a voluntary basis (Table 1) in the mandatory sample of 2015 and compared it with the results of the other 11 hospitals that had not participated before the introduction of the mandatory recording. To assess possible differences, we used Mann–Whitney *U* tests. We conducted tests for F0 diagnosis and all other diagnoses taken together separately.

**Table 1.** Number of hospitals and treated cases.

Year	Hospitals (*N*)	Cases (*N*)	Cases with F0, *N* (%)
2004	10	36,690	3,572 (9.7%)
2005	8	23,944	2,370 (9.9%)
2006	6	16,805	1,635 (9.7%)
2007	14	51,652	4,887 (9.5%)
2008	10	29,998	3,244 (10.8%)
2009	11	38,975	3,853 (9.9%)
2010	12	42,239	4,246 (10.1%)
2011	15	54,974	5,110 (9.3%)
2012	15	59,029	6,030 (10.2%)
2013	15	57,450	5,946 (10.3%)
2014	18	75,343	6,724 (8.9%)
2015	29	97,936	8,723 (8.9%)
2016	31	107,243	8,919 (8.3%)
2017	31	114,799	9,387 (8.2%)
2018	31	113,146	8,957 (7.9%)
2019	31	118,016	10,258 (8.7%)
**Total**		**1,038,239**	**96,381 (9**.**0%)**

## Results

Mann–Whitney *U* tests for differences in the proportions of cases involving coercive interventions showed no differences between hospitals that had participated in 2014 and the other hospitals in the mandatory sample of 2015 (all *p* > 0.05). We also found no differences in the cumulated duration of coercive measures (all *p* > 0.05). Therefore, we assume that the samples of hospitals in the years from 2004 to 2014 should be approximately representative, while from 2015 to 2019 all psychiatric hospitals in the Federal State of Baden-Wuerttemberg are covered in a full survey.

**Figure 1.**
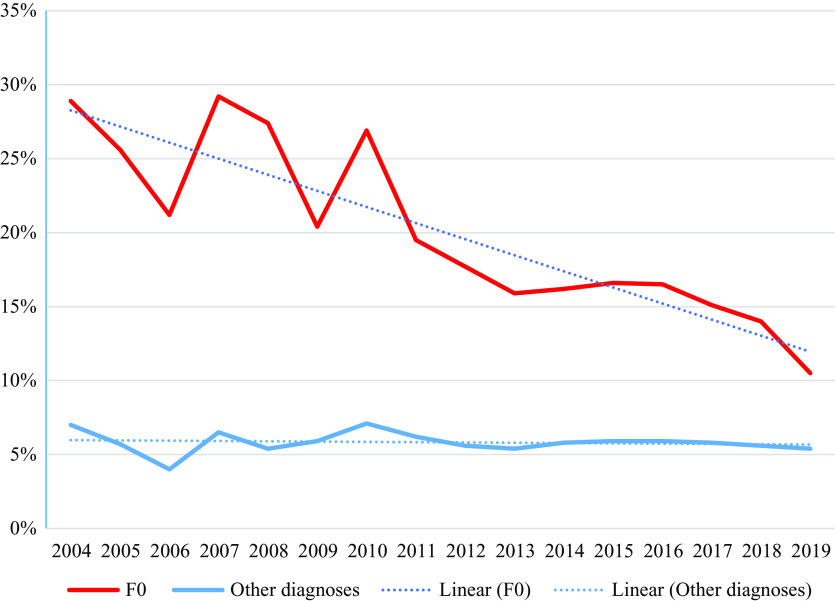
Proportion of cases with coercive interventions 2004–2019.

**Figure 2.**
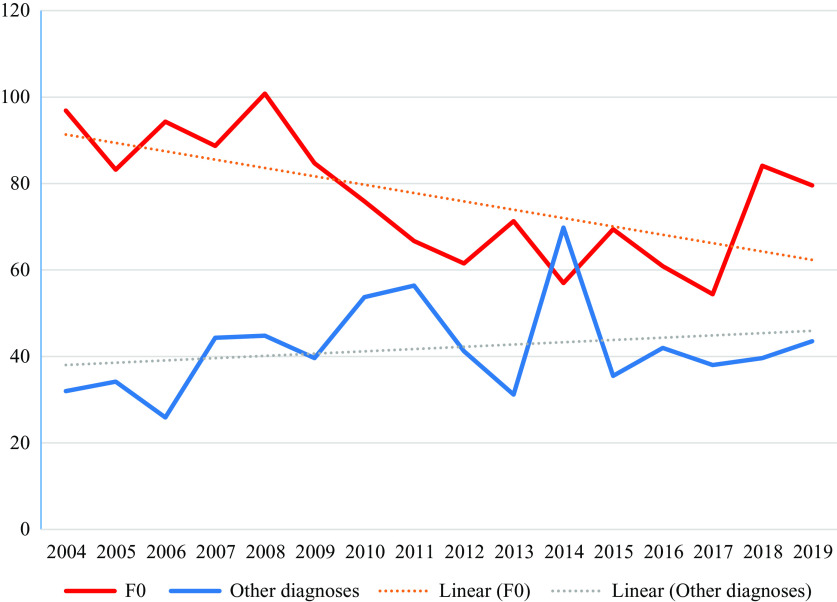
Cumulated duration (hours) of coercive interventions per affected case 2004–2019.

For the years 2004–2019, we analyzed data of a total of 1,038,239 cases, of which 93,861 (9.0%) had a diagnosis of an ICD-10 F0 disorder (Table 1). During this period, the proportion of cases affected by coercive interventions decreased from 28.4 to 10.5% in patients with F0 disorders, while rates in patients with other diagnoses decreased from 7.0 to 5.4%. Details for diagnostic groups according to ICD-10 are displayed in Table 2. The cumulated duration of coercive interventions per affected case dropped in patients with F0 diagnosis from 96.9 to 79.6 h but increased from 32.0 to 43.5 h in patients with other diagnoses. Details for diagnostic groups according to ICD-10 are displayed in Table 3. Figure 1 shows that the proportion of cases affected by coercive measures dropped continuously in cases with an F0 diagnosis, while it remained roughly constant for the remaining patients with all other diagnoses. Figure 2 shows that the mean duration of coercive measures per affected case decreased in patients with F0 diagnoses, while it even increased in the remaining patients with all other diagnoses. Regression analyses indicate that the decrease in the proportion of cases with coercive interventions (*b* = −1.0 and *p* < 0.01) as well as the decrease in the cumulated duration of coercive interventions (*b* = −2.4 and *p* < 0.01) are statistically significant in patients with F0 diagnosis. Regression analyses for other than F0 diagnoses revealed no statistically significant changes (all *p* ≥ 0.05). Visual inspection of the plots of observed versus predicted values revealed no substantial deviations from linearity. Visual inspection of the plots of residuals versus time showed no substantial violation of homoscedasticity. Kolmogorov–Smirnov tests indicated no significant deviation of residuals from normal distribution (all *p* ≥ 0.05).

**Table 2.** Proportions of cases with coercive interventions for the different diagnoses.

	**Proportion of cases with coercive interventions** **Mean (SD)**									
**Year**	**F0**	**F1**	**F2**	**F3**	**F4**	**F5**	**F6**	**F7**	**F8**	**F9**
2004	28.9% (14.8%)	4.5% (3.1%)	15.5% (4.3%)	3.1% (1.4%)	2.5% (1.6%)	n.a.	5.0% (5.0%)	n.a.	n.a.	n.a.
2005	25.6% (13.4%)	3.1% (1.3%)	12.4% (3.3%)	3.4% (1.7%)	1.8% (1.5%)	n.a.	6.8% (6.8%)	n.a.	n.a.	n.a.
2006	21.2% (8.0%)	3.7% (1.8%)	11.9% (4.1%)	3.7% (1.3%)	2.0% (0.9%)	n.a.	4.3% (4.3%)	n.a.	n.a.	n.a.
2007	29.2% (16.4%)	4.4% (3.0%)	14.0% (4.8%)	3.8% (1.7%)	2.5% (1.9%)	n.a.	4.0% (4.0%)	n.a.	n.a.	n.a.
2008	27.4% (20.4%)	3.5% (1.9%)	11.8% (3.8%)	2.8% (1.4%)	2.3% (2.3%)	n.a.	3.9% (3.9%)	n.a.	n.a.	n.a.
2009	20.4% (7.7%)	3.2% (1.5%)	12.1% (4.9%)	2.6% (1.1%)	2.7% (2.4%)	n.a.	9.2% (9.2%)	n.a.	n.a.	n.a.
2010	26.9% (18.3%)	5.3% (5.0%)	13.1% (4.5%)	3.2% (1.7%)	2.8% (1.9%)	n.a.	7.6% (7.6%)	n.a.	n.a.	n.a.
2011	19.5% (7.7%)	4.3% (4.3%)	13.4% (5.2%)	3.4% (2.0%)	2.9% (3.0%)	n.a.	5.5% (5.5%)	n.a.	n.a.	n.a.
2012	17.7% (8.9%)	4.4% (4.7%)	14.1% (4.5%)	2.7% (1.5%)	2.2% (1.8%)	n.a.	4.9% (4.9%)	n.a.	n.a.	n.a.
2013	15.9% (7.4%)	3.8% (3.9%)	14.0% (5.1%)	2.8% (1.8%)	3.5% (4.0%)	n.a.	6.1% (6.1%)	n.a.	n.a.	n.a.
2014	16.2% (8.8%)	4.0% (3.5%)	13.8% (4.7%)	3.1% (2.3%)	4.1% (8.2%)	n.a.	3.8% (3.8%)	n.a.	n.a.	n.a.
2015	16.6% (9.1%)	4.7% (6.5%)	15.0% (5.6%)	2.4% (1.5%)	2.5% (2.2%)	2.7% (12.8%)	7.3% (5.1%)	14.3% (16.8%)	9.9% (27.2%)	3.8% (9.9%)
2016	16.5% (8.5%)	4.7% (6.6%)	14.1% (5.7%)	2.1% (1.3%)	2.8% (1.9%)	2.1% (5.8%)	7.8% (7.4%)	20.6% (17.0%)	7.8% (12.6%)	4.5% (21.3%)
2017	15.1% (7.8%)	5.3% (4.6%)	14.4% (5.3%)	2.1% (1.1%)	3.1% (1.8%)	2.5% (7.0%)	9.4% (5.8%)	23.2% (17.3%)	8.4% (23.4%)	4.9% (8.7%)
2018	14.0% (10.9%)	5.1% (5.8%)	13.7% (4.9%)	2.2% (1.0%)	2.9% (1.9%)	0.9% (4.0%)	9.0% (7.4%)	24.6% (27.0%)	13.5% (18.3%)	5.2% (8.7%)
2019	10.5% (8.6%)	4.5% (8.7%)	13.7% (5.5%)	2.1% (2.1%)	2.8% (2.1%)	1.5% (4.1%)	9.0% (10.3%)	20.8% (20.9%)	11.6% (19.6%)	4.0% (19.2%)

Abbreviation: n.a., not available; SD, standard deviation.

**Table 3.** Cumulated duration of coercive interventions per affected case for the different diagnoses.

	**Cumulated duration of coercive interventions per affected case (hours)** **Mean (SD)**									
**Year**	**F0**	**F1**	**F2**	**F3**	**F4**	**F5**	**F6**	**F7**	**F8**	**F9**
2004	96.9 (62.8)	23.2 (13.1)	37.2 (14.8)	32.7 (13.5)	24.6 (44.9)	n.a.	14.2 (7.9)	n.a.	n.a.	n.a.
2005	83.2 (70.7)	12.0 (5.8)	39.6 (26.4)	27.6 (19.3)	18.7 (25.8)	n.a.	30.4 (21.2)	n.a.	n.a.	n.a.
2006	94.3 (65.0)	26.3 (30.2)	29.3 (14.5)	40.1 (29.5)	15.9 (16.6)	n.a.	36.0 (28.0)	n.a.	n.a.	n.a.
2007	88.7 (61.5)	30.0 (43.1)	43.6 (21.0)	43.4 (32.6)	12.7 (7.8)	n.a.	31.4 (24.1)	n.a.	n.a.	n.a.
2008	100.8 (88.9)	27.9 (28.8)	36.4 (21.2)	35.7 (29.7)	22.3 (27.0)	n.a.	31.1 (27.8)	n.a.	n.a.	n.a.
2009	84.7 (84.6)	14.1 (7.2)	42.1 (16.6)	23.1 (12.2)	12.5 (9.8)	n.a.	53.0 (35.5)	n.a.	n.a.	n.a.
2010	75.9 (62.8)	21.7 (19.1)	42.9 (19.2)	39.2 (25.4)	27.6 (40.5)	n.a.	33.8 (35.6)	n.a.	n.a.	n.a.
2011	66.7 (50.8)	15.4 (11.1)	48.9 (17.4)	26.9 (20.4)	28.7 (54.1)	n.a.	19.1 (12.4)	n.a.	n.a.	n.a.
2012	61.5 (52.8)	17.0 (14.2)	42.6 (20.3)	35.5 (18.7)	22.6 (13.8)	n.a.	27.9 (35.3)	n.a.	n.a.	n.a.
2013	71.3 (46.0)	12.5 (8.3)	40.4 (14.8)	35.0 (28.5)	25.3 (33.0)	n.a.	52.6 (51.0)	n.a.	n.a.	n.a.
2014	57 (46.1)	16.5 (15.5)	46.1 (23.4)	28.7 (17.4)	18.7 (16.9)	n.a.	41.6 (85.5)	n.a.	n.a.	n.a.
2015	69.4 (29.3)	18.0 (32.7)	47.2 (15.2)	32.2 (18.8)	16.9 (21.9)	23.3 (4.3)	30.5 (18.7)	35.5 (10.8)	110.8 (15.5)	30.2 (33.4)
2016	60.9 (30.5)	17.8 (13.5)	59.3 (31.1)	43.2 (31.4)	15.6 (10.2)	21.1 (25.5)	34.6 (17.1)	48.9 (7.6)	58.8 (40.6)	12.7 (5.7)
2017	54.4 (36.1)	18.9 (19.4)	52.6 (40.5)	36.0 (12.1)	16.9 (7.5)	28.6 (19.2)	32.0 (6.3)	60.4 (19.8)	21.8 (6.4)	16.3 (5.9)
2018	84.1 (129.5)	19.1 (20.1)	52.8 (26.0)	40.7 (18.0)	21.4 (9.8)	27.6 (2.8)	38.7 (18.4)	82.3 (14.9)	22.1 (9.2)	10.9 (8.9)
2019	79.6 (60.3)	21.6 (25.3)	53.8 (25.0)	48.9 (25.0)	20.6 (11.7)	26.1 (5.3)	35.2 (18.1)	91.2 (19.4)	222.9 (56.9)	8.7 (6.8)

Abbreviation: n.a., not available; SD, standard deviation.

## Discussion

The results provide good news and bad news. The good news is that the use of coercive interventions in old age psychiatry has dropped by about 50% under real-world conditions. A reduction of serious unwanted outcomes to such a degree in routine care is remarkable and rather uncommon not only in psychiatry but also in healthcare in general. The bad news is that the continuous reduction of the percentage of admissions affected by seclusion or restraint that we had reported in a previous study [24] disappears if organic disorders are separated from the analysis. In general psychiatry, notwithstanding a considerable number of evidence-based interventions that have been recommended in guidelines and a supposed increasing awareness, a substantial effect cannot be demonstrated so far. Reasons might be that guideline-based recommendations have still not yet been sufficiently implemented in clinical practice, or, at least theoretically but not supported by evidence, that the proportion of admissions with severe behavior problems could have increased, or that the use of coercion is at such a low level that further reductions are difficult to achieve. Another reason could be the different baseline in both groups (28.9 vs. 7.0% affected by coercive measures) and a greater potential for improvement in the group of patients with organic disorders.

In old age psychiatry, evidence has been increasing that mechanical restraint is not effective in preventing falls and reduction of restraint is not associated with an increase of falls [19,25]. Though not supported by strong evidence, technical devices designed to prevent falls or to mitigate their consequences such as hip protectors [26–28], low–low beds [29], bed–chair pressure sensors [30], gait-stabilizing devices [31], as well as physical training [32,33] suggest promising alternatives to freedom-restricting interventions. Moreover, frequent awareness workshops and conferences for physicians and nurses have emphasized the necessity to reduce coercive measures. Several high court decisions and consecutive changes in legislation, though not directed at nursing in old age psychiatry in particular, strengthened the sensibility with respect to violating patient’s integrity and freedom [34]. Now, there is evidence that these interventions have successfully changed clinical practice. Nowadays, the use of seclusion or restraint except for seriously violent behavior and some exceptional cases of self-endangerment is increasingly considered as malpractice.

In contrast, it seems disappointing that many unsystematic efforts to introduce evidence-based interventions to reduce coercion in general psychiatry have not yielded significant effects in broad clinical practice, pointing to the well-known gap between efficacy in well-designed clinical trials and effectiveness in routine care [35]. More specific and systematic implementation strategies are required. Their efficacy is currently being tested in a randomized controlled trial with 52 participating wards [36]. An alternative explanation for the obvious difficulty to reduce coercion in general psychiatry could be an increase of patients exhibiting violent behavior over time. This is frequently claimed among clinicians and nurses. However, there is no evidence, neither from Germany nor from other European countries.

This study has some limitations. First, the validity of routine data could be questioned. However, for the case registry, the validity has been examined and has proved to be good [20] with data extracted directly from patients’ electronic charts. A small minority of smaller hospitals still uses paper documents, implicating some risk of under-reporting. Second, validity and representativity of the data obtained in 2004–2014 before the mandatory implementation of the registry could be put into question. In this period, only about a third up to a half of the State’s psychiatric hospitals had participated on a voluntary basis. However, representativity should be sufficient as we double-checked that those hospitals participating in 2014 on a voluntary basis did not differ significantly in 2015 from the other hospitals that had then been obligated to join by law. Third, a source of weakness of the data could be that until 2014 data were delivered at an aggregated level by hospitals themselves while since 2015 raw data have been delivered to the central registry and can be directly extracted from the charts by the data evaluation office. However, since percentages of cases exposed to coercive interventions are nearly identical in 2014 and in 2015, we consider this source of bias negligible. Fourth, the strict regulations concerning data privacy of the Federal State’s Data Security Officer limit available information to the characteristics and duration of the coercive intervention, and patient’s diagnosis and gender. Linking with further patient variables and multilevel statistical analyses are not possible. Lastly, patient populations probably changed in the course of 15 years, regulating laws underwent some changes, and so did the structure of psychiatric hospitals. For example, the number of places in day clinics as well as in psychotherapeutic departments has increased, and the same applies for outpatient services. Therefore, the results obtained from this longitudinal observational study need to be interpreted with caution.

## Conclusion

In conclusion, the insight gained from this study indicates that healthcare copes more easily with risks toward self due to frailty than with risk toward others due to violence. The latter is still an open challenge. As a considerable range of evidence-based interventions for the prevention of coercion and violence is available now, further research should try to improve implementation of these interventions in routine care and evaluate the effectiveness of programs targeted at this purpose in general and especially for people with organic disorders or cognitive impairment to further reduce the use of coercion.

## Data Availability

The data that support the findings of this study are available from the Ministerium für Soziales und Integration, Baden-Wuerttemberg, Ombudsstelle: https://sozialministerium.baden-wuerttemberg.de/de/gesundheit-pflege/medizinische-versorgung/psychiatrische-versorgung/unabhaengige-anlaufstellen/. Restrictions apply to the availability of these data, which were used under license for this study. Data are available from the authors with the permission of the Ministery.
